# *In Vivo* Activity of Antimicrobial Peptoid Oligomers against HSV‑1 in a Mouse Model of Herpes Labialis

**DOI:** 10.1021/acsinfecdis.6c00224

**Published:** 2026-07-05

**Authors:** Erika L. Figgins, Denny Gao, Lisa K. Ryan, Jennifer S. Lin, Natalia Molchanova, Edward M. Rudnic, Sheetal Vali, Brian S. Shumway, Kent Kirshenbaum, Annelise E. Barron, Gill Diamond

**Affiliations:** † Department of Oral Immunology and Infectious Diseases, University of Louisville School of Dentistry, Louisville, Kentucky 40202, United States; ‡ Center for Predictive Medicine, 5170 University of Louisville, Louisville, Kentucky 40202, United States; § Department of Bioengineering, 6429 Stanford University Schools of Medicine and Engineering, Stanford, California 94305, United States; ∥ Molecular Foundry, Lawrence Berkeley National Laboratory, Berkeley, California 94720, United States; ⊥ Maxwell Biosciences, Austin, Texas 78738, United States; # Department of Diagnosis and Oral Health, 162193 University of Louisville School of Dentistry, Louisville, Kentucky 40202, United States; ⊗ Department of Chemistry, 5894 New York University, New York, New York 10003, United States

**Keywords:** Antimicrobial peptides, antiviral, herpes labialis, cathelicidin

## Abstract

Herpes simplex virus type-1 (HSV-1) infections cause recurrent oral lesions and are the primary cause of infectious blindness and genital lesions in developed countries. HSV-1 can also lead to life-threatening infections in immunocompromised individuals. Currently, the primary class of antiviral therapeutics for HSV-1 treatment are nucleoside analogues such as acyclovir. However, these therapeutics are only partially effective and are subject to development of resistance. Thus, the development of new, effective antiviral agents, which can be used topically to inactivate HSV-1, is a necessity. We have recently demonstrated the potent antiviral activity of a family of biomimetic compounds. These ‘peptoid’ (*N*-substituted glycine) oligomers mimic the structure and activity of antimicrobial peptides like LL-37, while being highly resistant to proteases. Antiviral peptoids rapidly inactivate HSV-1 *in vitro* by disruption of the viral envelope, likely through interaction with the lipid phosphatidylserine on the outer membrane leaflet. To test the *in vivo* activity of these peptoids, we optimized a lip treatment model of HSV-1 infection. We demonstrate that delivery of a clinical isolate of HSV-1, strain 294.1, onto scarified lips of BALB/c mice led to reproducible lesions within 5 days. Treatment of these lesions on day 2 post infection with increasing concentrations of an antiviral peptoid resulted in a dose-dependent inhibition of lesion formation, comparable to acyclovir cream, and the host defense peptide LL-37. The same dose-dependence was observed when quantifying viral DNA from both the lip and the trigeminal ganglion by qPCR. The results demonstrate the ability of these peptoids to be developed as topical antiviral agents to prevent herpes labialis infections caused by HSV-1.

## Graphical abstract

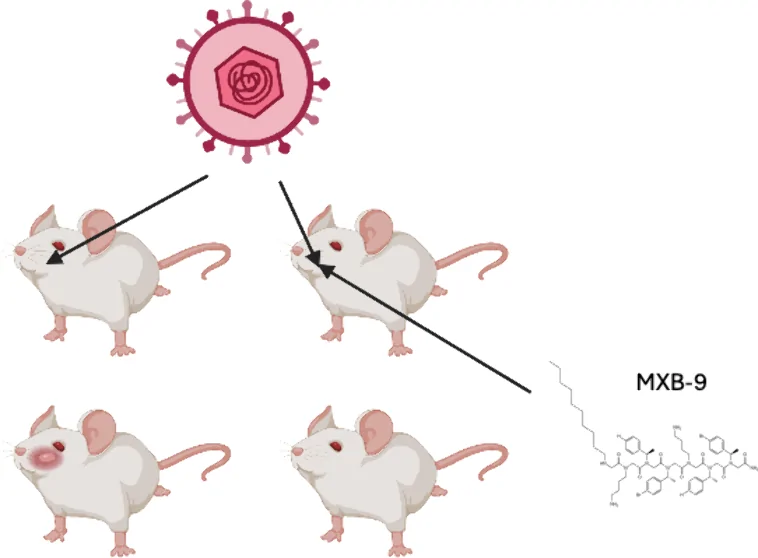

## Introduction

We recently described the potent, rapid *in vitro* antiviral activity of a class of antimicrobial peptide mimics, called peptoids, against herpes simplex virus-1 (HSV-1) and SARS-CoV-2.^1^ This activity is dose- and time-dependent and leads to the disruption of the viral envelope. Furthermore, the peptoids show no cytotoxicity when tested against primary cultures of epithelial cells. These results suggested that the peptoids could be developed as topical antivirals for lip infections of HSV-1.

HSV-1 infects a substantial portion of the world’s population, estimated to be over two-thirds of adults under the age of 50.^2^ It primarily manifests as oral lesions but can also lead to more severe conditions such as herpes simplex keratitis, a leading cause of infectious blindness, and herpes encephalitis, a potentially life-threatening brain inflammation (reviewed in ref^3^). Beyond these immediate health concerns, HSV-1’s ability to establish lifelong latency in sensory neurons poses ongoing challenges for the development of effective treatments.^4^

Current treatments for HSV-1, such as acyclovir, have been the mainstay in managing this infection. Acyclovir and its derivatives function by disrupting the viral replication cycle, specifically by inhibiting the viral DNA polymerase, which is crucial for viral DNA synthesis.^5^ While these drugs have provided substantial relief to individuals with HSV-1 infections, they have their own limitations. Acyclovir is primarily effective against actively replicating viruses, which means that it may not address latent infections where the virus remains dormant within sensory neurons, such as the trigeminal ganglia. Furthermore, the prolonged and widespread use of acyclovir has led to the emergence of drug-resistant strains of HSV-1.^6^ This underscores the pressing need for an alternative therapeutic approach.

Antimicrobial peptides (AMPs) have been examined as potential therapeutics to combat these issues.^7^ Among these AMPs, the human cathelicidin peptide LL-37 has emerged as a promising candidate. LL-37 is a naturally occurring peptide found in various cells and tissues of the human body. It exhibits broad-spectrum antimicrobial activity capable of combating bacteria, fungi, and viruses.^8^ Studies have demonstrated its ability to breach microbial membranes, including viral envelopes,^9^ offering a potential avenue for inhibiting HSV-1 infectivity.

However, the clinical application of LL-37 and other AMPs has been hampered by challenges, such as cost, susceptibility to protease cleavage, and limited bioavailability. These limitations have driven researchers to explore alternatives, leading to the investigation of antimicrobial peptoids as a novel class of compounds with potential advantages over traditional AMPs.^10^ Antimicrobial peptoids (sequence-specific oligo-*N*-substituted glycines) are characterized by their altered chemical structures as compared to peptides, which offer improved bioavailability and stability compared with natural AMPs. While a peptoid is based on a similar polyamide backbone as natural peptides, the side chains are appended to the backbone nitrogen.^10^ We have designed numerous peptoids with similar properties as LL-37, including the ability to self-assemble into micelles in a physiological environment, enhancing their ability to disrupt microbial membranes.^11^ Our previous studies demonstrated the *in vitro* antiviral activity of these peptoids against HSV-1 through membrane interactions, with no observable cytotoxicity when tested against primary cultures of oral keratinocytes.^1,12^

This study aims to define the mechanism of action of these antiviral peptoids and assess the activity of the most potent peptoids in a mouse model of HSV-1 labial infection. While previous studies have demonstrated limited infection of laboratory strains in mice, the current models were insufficient to quantify *in vivo* activity accurately. Here, we demonstrate a robust mouse model for HSV-1 lip infection and show the potent *in vivo* activity of an antimicrobial peptoid when delivered topically after infection.

## Results

### Structure/Function Comparison of Activity of Peptoids and LL-37

Based on our previous *in vitro* study,^1^ we chose to further examine the antiviral capability of the peptoid heptamer MXB-9 ^13^ (Figure 1A), which mimics the essential structural elements of LL-37 ^14^ (amphipathic with cationic and hydrophobic side chains) while incorporating a C10 tail inspired by lipopeptides.^15^ When tested against HSV-1 *in vitro*, LL-37 demonstrates an IC_50_ of 9.8 μg/mL (Figure 1B), which is comparable to the of 8.8 μg/mL for MXB-9 (Figure 1C) . In a parallel experiment, we measured the effect of MXB-9 on viable HSV-1 by quantifying TCID_50_, which shows a similar response (Figure 1D).

**Figure 1:**
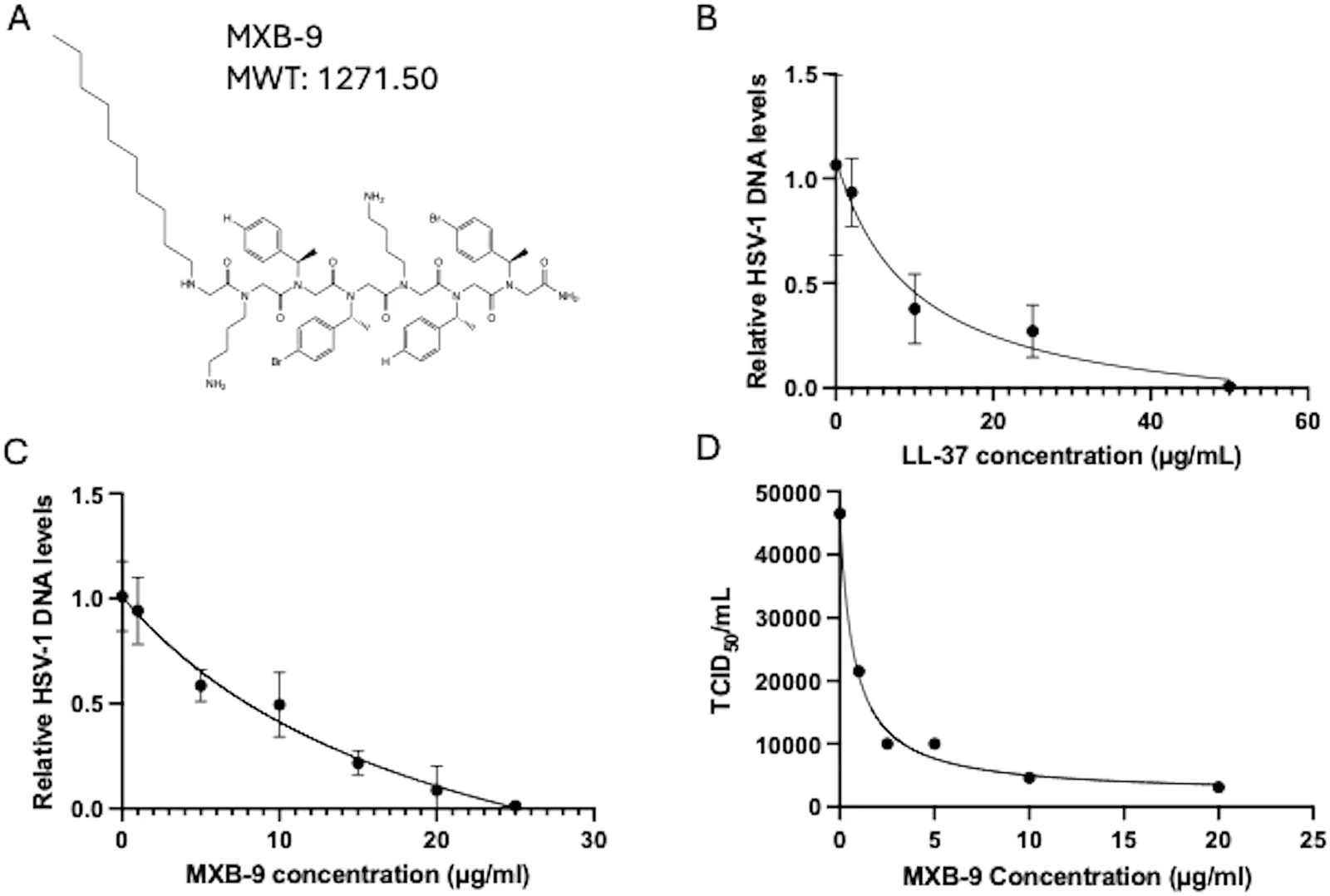
Structure MXB-9 and *in vitro* antiviral activity of MXB-9 and LL-37. (A) MXB-9 is a sequence-specific oligo-*N*-substituted glycine heptamer. *In vitro* antiviral activity of LL-37 (B) and MXB-9 (C) were assessed by qPCR of HSV-1 DNA as described previously.^1^ The effect of MXB-9 on viable virus was quantified by TCID_50_ analysis (D). Activity was calculated by nonlinear regression analysis (GraphPad).

### Mechanism of Action of Antimicrobial Peptoids against HSV-1

Initial studies suggested that the antiviral peptoids targeted the viral envelope.^1,12,16^ To further investigate these findings, we examined the activity of MXB-9 at each stage of the HSV-1 viral life cycle. To achieve this, human telomerase-immortalized gingival keratinocytes (TIGK) cells were infected at various time points and temperatures to isolate the different stages of the HSV-1 life cycle. A citric acid wash was employed to inactivate noninternalized viruses and remove any remaining extracellular viral particles. Figure 2A illustrates a significant reduction in HSV-1 DNA levels at the preattachment stage, with MXB-9 showing a 6.25-fold reduction compared to the untreated control. No significant reduction was seen in other stages, including viral attachment, penetration, and postentry. These results support our hypothesis, based on the membrane disruption observed,^1^ that this peptoid exhibits predominantly extracellular antiviral activity in cultured cells. Since this mechanism is distinct from the intracellular mechanism of acyclovir, we tested the activity of MXB-9 in the presence and absence of acyclovir. We determined that the addition of 0.3 μg/mL acyclovir was sufficient to reduce the IC_50_ of MXB-9 by a factor of 2.2 (Figure 2B).

**Figure 2:**
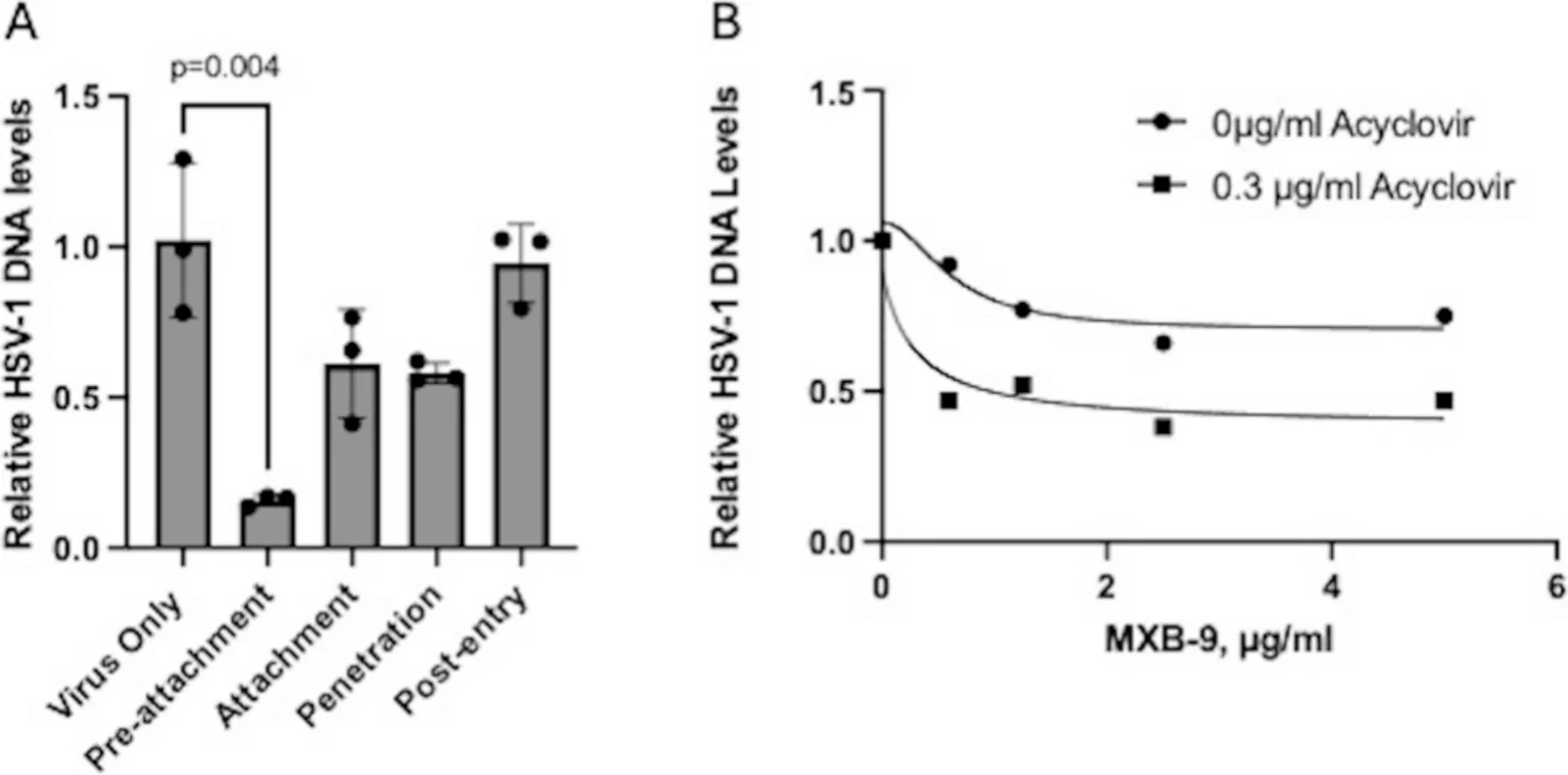
Mechanism of action of MXB-9. (A) HSV-1-GFP virus was incubated with MXB-9 (20 μg/mL) at different points in the HSV-1 life cycle. 24 h postinfection total DNA was isolated and HSV-1 DNA levels were quantified by qPCR. Virus was incubated with peptoid either prior to infection (“pre-attachment”); during a 4 °C incubation (“attachment”); after removing extracellular virus with citric acid (“penetration”); or after entry (“post-entry. Statistical significance was determined by two-tailed t-test compared with untreated virus-infected control (*n* = 3). (B) synergistic activity of MXB-9 with acyclovir. HSV-1-GFP was incubated with Vero cells in the presence of increasing concentrations of MXB-9 in the presence or absence of 0.3 μg/mL acyclovir for 2 h. Medium was replaced with fresh medium without antivirals and incubated for a further 24 h. Total DNA was isolated and HSV-1 DNA was quantified as above. IC_50_ was calculated by nonlinear regression (GraphPad).

### Optimization of the In Vivo Model

#### Selection of Optimal Mouse Strain

Initial examination of a published lip scarification model of herpes labialis^17^ suggested it would not provide a sufficiently robust assay system to test the efficacy of a potential new therapeutic entity (data not shown), most likely due to the use of C57Bl/6 mice, which are less susceptible to herpesvirus infections.^18^ To optimize the mouse model for HSV-1 infection and the manifestation of visible lesions, we examined three mouse strains that have been previously acknowledged as effective models for HSV-1 through oral infection, and which led to dissemination to the trigeminal ganglia.^19^ BALB/c, A/J, and SJL/J strains (*n* = 5 per group) were compared to C57BL/6 mice using the lip scarification technique infected with HSV-1 GFP. On the basis of these results, we observed lesions for each strain, aside from C57BL/6, at day 5. In Figure 3, no visible lesions were observed throughout the entire 5-day infection period in the C57Bl/6 mice. However, in the A/J and SJL/J groups, visible lesions were identified in 80% and 60% of the mice, respectively. Notably, 100% of the BALB/c mice exhibited visible and well-developed lesions. In light of these findings, BALB/c mice were chosen for subsequent experiments to further advance the development of an *in vivo* mouse model for HSV-1 infection and the formation of visible lesions.

**Figure 3:**
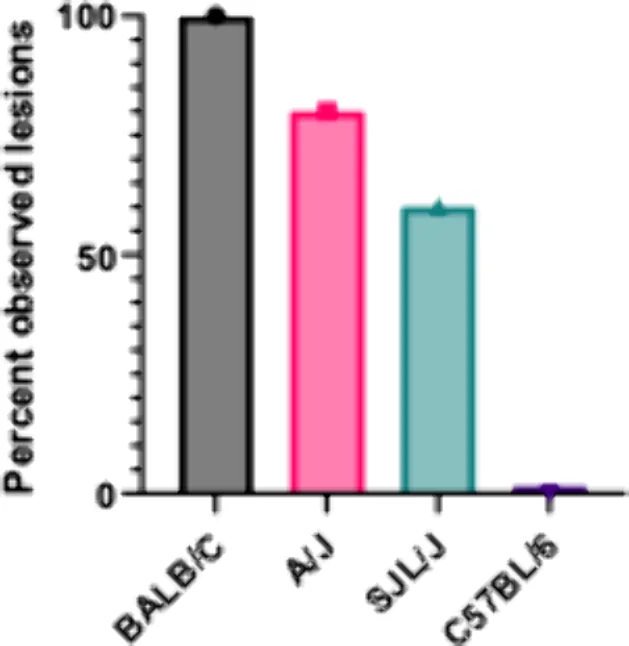
Assessment of mouse strains for HSV-1 Infection in the mouse model. BALB/c, A/J, SJL/J, or C57BL/6 mice (*n* = 5 per group) were subjected to infection with HSV-GFP using the lip scarification model. The percent of mice displaying observable lesions over a 5-day infection period is shown.

#### HSV-1 Strain Susceptibility In Vitro

To determine the optimal strain of virus for the *in vivo* model, we first tested the susceptibility of different strains of HSV-1 to MXB-9 *in vitro*. All strains were tested with 20 μg/mL MXB-9, based on data from our previous study.^1^ These included both acyclovir-sensitive (KOS and 294.1) and resistant (PAAr5, TKR, 615.8, and 615.9) strains. As seen in Figure 4, all strains demonstrated similar susceptibility to the peptoid, although KOS, 294.1 and PAAr5 exhibited the most sensitivity, with complete viral inhibition seen at this concentration.

**Figure 4:**
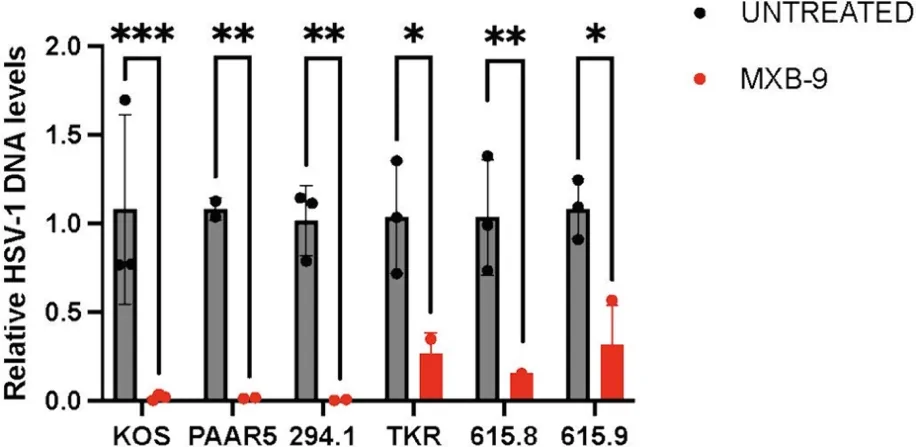
*In vitro* activity of antimicrobial peptoid against acyclovir-sensitive and resistant HSV-1 strains. Activity of MXB-9 was tested in an *in vitro* assay at 20 μg/mL for 2 h against six HSV-1 strains (PAAr5, TKR, KOS, 615.8D, 615.9, 294.1). HSV-1 DNA levels were quantified by qPCR. Significance was determined by 2-tailed *t* test. *, *p* < 0.05; **, *p* < 0.005; ***, *p* < 0.0005. Bars represent the standard deviation. (*n* = 3 per strain and condition).

#### HSV-1 Strain Susceptibility in BALB/c Mice

To determine the severity of lesions caused by different strains of HSV-1, BALB/c mice (*n* = 5 per group) were infected with strains of HSV-1 (GFP, KOS, 294.1, PAAr5) using the lip abrasion model. In this model, lesions begin to appear on day 3 postinfection (pi), reaching a maximum size between day 5–6, and resolve on their own by 8 days pi. To quantify lesion size, we observed mice daily for lesion development on days three, four, and five pi. The lesion diameters were quantified after photomicroscopy (Figure 5A). On day 5 pi, the lips were harvested and then homogenized, and the homogenate was used to determine the recoverable viral titer by a plaque assay (Figure 5B). Infection with strain PAAr5 produced small lesions with only 2 mice having lesions on day 4 that resolved by day 5. Also, only one mouse had recoverable virus of 1 × 10^6^ pfu/g tissue, and this mouse did not have any visible lesion development. Infection with KOS resulted in only 2 mice that developed observable lesions; however, viral titers were recoverable from these same mice (1 × 10^7^ and 2 × 10^5^ pfu/g). HSV-GFP had slightly more consistent infection outcomes with 3 mice developing lesions, but overall, smaller lesions and lower viral titers (2 × 10^4^ pfu/g). However, strain 294.1 presented the largest and most consistent lesions. Only one mouse did not develop a lesion over the 5 days, with no recoverable virus. The other four mice had lesions that ranged in size from 0.5 to 3.0 mm. The viral titers also ranged from 2 × 10^5^ to 3 × 10^7^ pfu/g. The increased efficacy of infection and lesion severity of HSV-1 294.1 informed our choice to use this strain in the further development of the peptoid mouse model. Further, the results show that the lesion size on day 5 in general correlates with viral titer.

**Figure 5:**
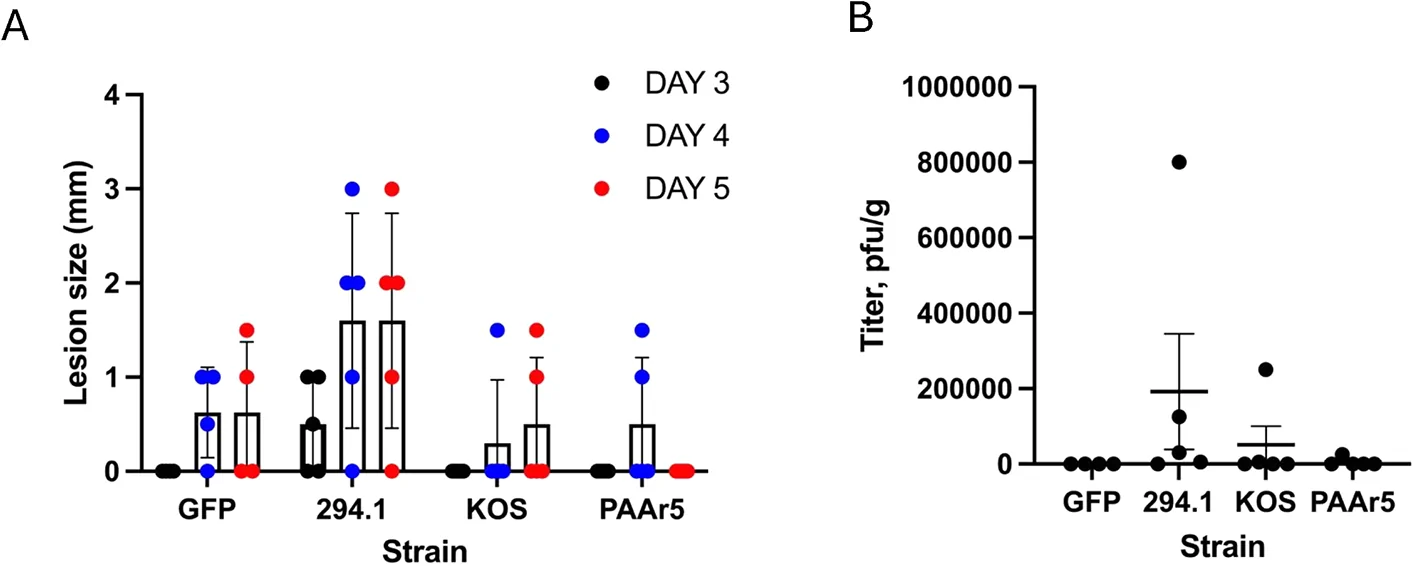
*In vivo* lesion size and viral titer by viral strain. Female BALB/c mice (*n* = 5 per group) were infected with 5 × 10^5^ pfu of different strains of HSV-1 by the lip scarification model. Lesions were photographed and diameter was measured on days 3–5 post infection (A). On day 5, mice were euthanized, lips were excised and homogenized in buffer. Viral titers were determined by plaque assay (B).

#### Test of Delivery Vehicle

To determine the optimal method of application of the antiviral agent in the model, we tested the ability of MXB-9 to inactivate HSV-1 in the presence of a number of potential delivery vehicles. We solubilized MXB-9 at 20 μg/mL in PBS as in a standard *in vitro* assay, or in combination with carboxymethyl cellulose (CMC), polyethylene glycol (PEG), polyvinylpyrrolidone (PVP) or poly­(ethylene oxide) (PEO). These combinations were incubated with HSV-1 for 2 h prior to infecting cells. The results shown in Figure 6 demonstrate that there was significantly diminished activity for all the potential vehicles compared with PBS alone. Therefore, we decided for this proof-of-concept study, to deliver the agent solely in PBS.

**Figure 6:**
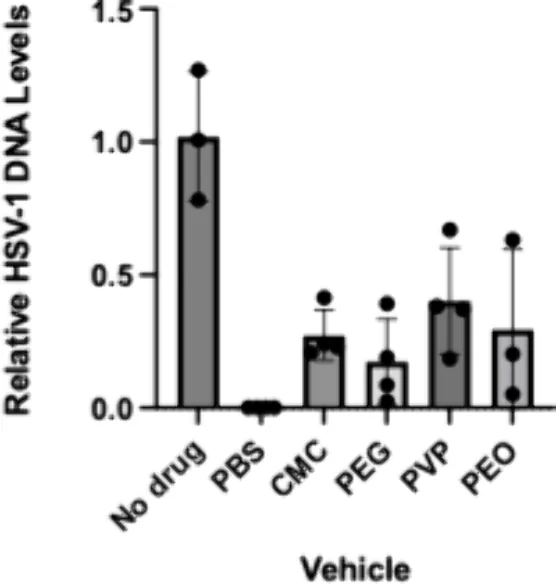
*In vitro* test of delivery vehicle effect on antiviral activity of MXB-9. MXB-9 was diluted to 20 μg/mL in either PBS alone, carboxymethyl cellulose (CMC), polyethylene glycol (PEG), polyvinylpyrillodone (PVP) or poly­(ethylene oxide) (PEO) and incubated with HSV-1 for 2 h prior to infection of cells (*n* = 3). Total DNA was isolated and quantified for HSV-1 DNA relative to β-actin.

#### Test of In Vivo Model

To demonstrate the ability to deliver an antiviral in this model, we first tested the topical application of LL-37. BALB/c mice were infected with the HSV-1 strain 294.1. For optimal infection, a chemical hair removal cream was applied 1 day before the virus infection on the mice’s lips to enhance lesion spread and visibility of smaller lesions. Lips were abraded, and the virus was applied as described in Materials and Methods. In preliminary experiments, lesion formation was not observed until day 3 (data not shown). Since this study was designed to examine the potential to prevent lesion formation, we chose to begin treating the infection on day 2 postinfection. When LL-37 was applied to the lips of infected mice at day 2 postinfection and then daily until day 5, we observed a significant reduction in lesion size on day 5 (Figure 7A), which was mirrored by a reduction in HSV-1 DNA levels in the lip (Figure 7B) and trigeminal ganglia (TG) (Figure 7C) to undetectable levels. Treatment also led to a similar reduction in viral titer as quantified by TCID_50_ analysis (Figure 7D,E).

**Figure 7:**
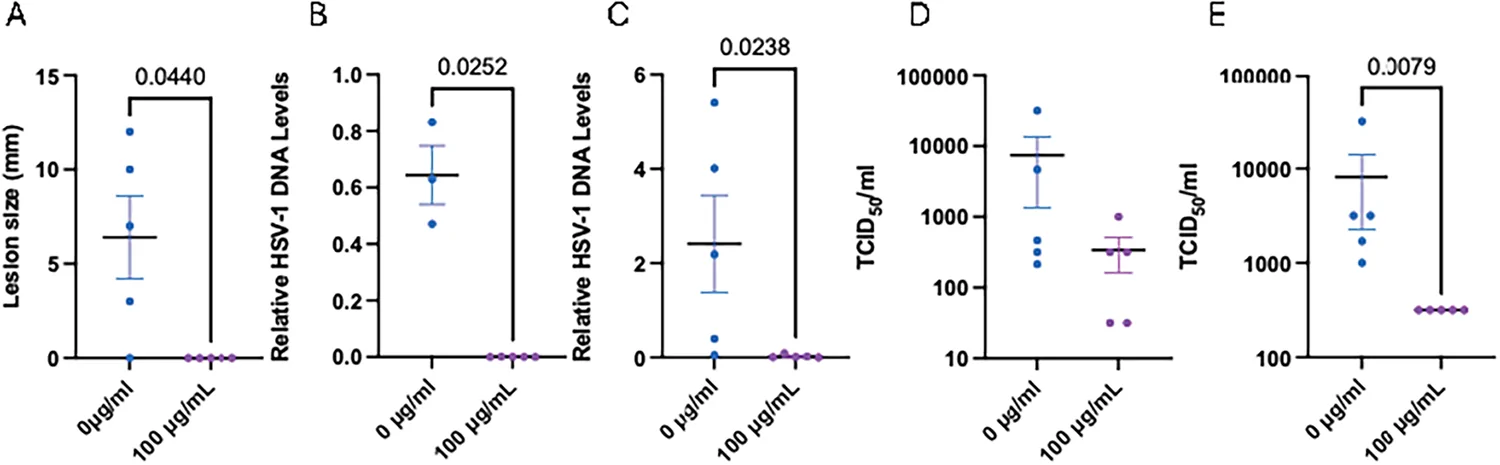
*In vivo* activity of LL-37 against HSV-1. BALB/c mice (*n* = 5 per group) were infected with HSV-1 strain 294.1 on day 0 and treated with 20 μL of 100 μg/mL of LL-37 in PBS on day 2 postinfection. Lips and TG were harvested on day 5 postinfection for DNA analysis. (A). Lesion size on day 5. HSV-1 DNA levels were quantified in excised lips (B) and trigeminal ganglia (C) by qPCR relative to mouse β-actin. Tissues were homogenized and TCID_50_ were quantified in lip (D) and TG (E) Statistical significance was evaluated using *t* test, with error bars representing the standard error of the mean. P-values are provided. Points represent individual animals.

#### In Vivo Activity of Peptoid

Mice were treated with 25, 50, and 100 μg/mL of MXB-9 in PBS or with PBS alone by directly applying the solution topically onto the lip on day 2 post infection, and then daily until day 5 postinfection. As a comparison with standard of care, we applied acyclovir cream (2%) in parallel. Lesions were photographed (Figure 8A) and lesion size was measured (Figure 8B) daily. On day 5 mice were euthanized, and lips and TG were excised, DNA was isolated and HSV-1 viral DNA was quantified by qRT-PCR.

**Figure 8:**
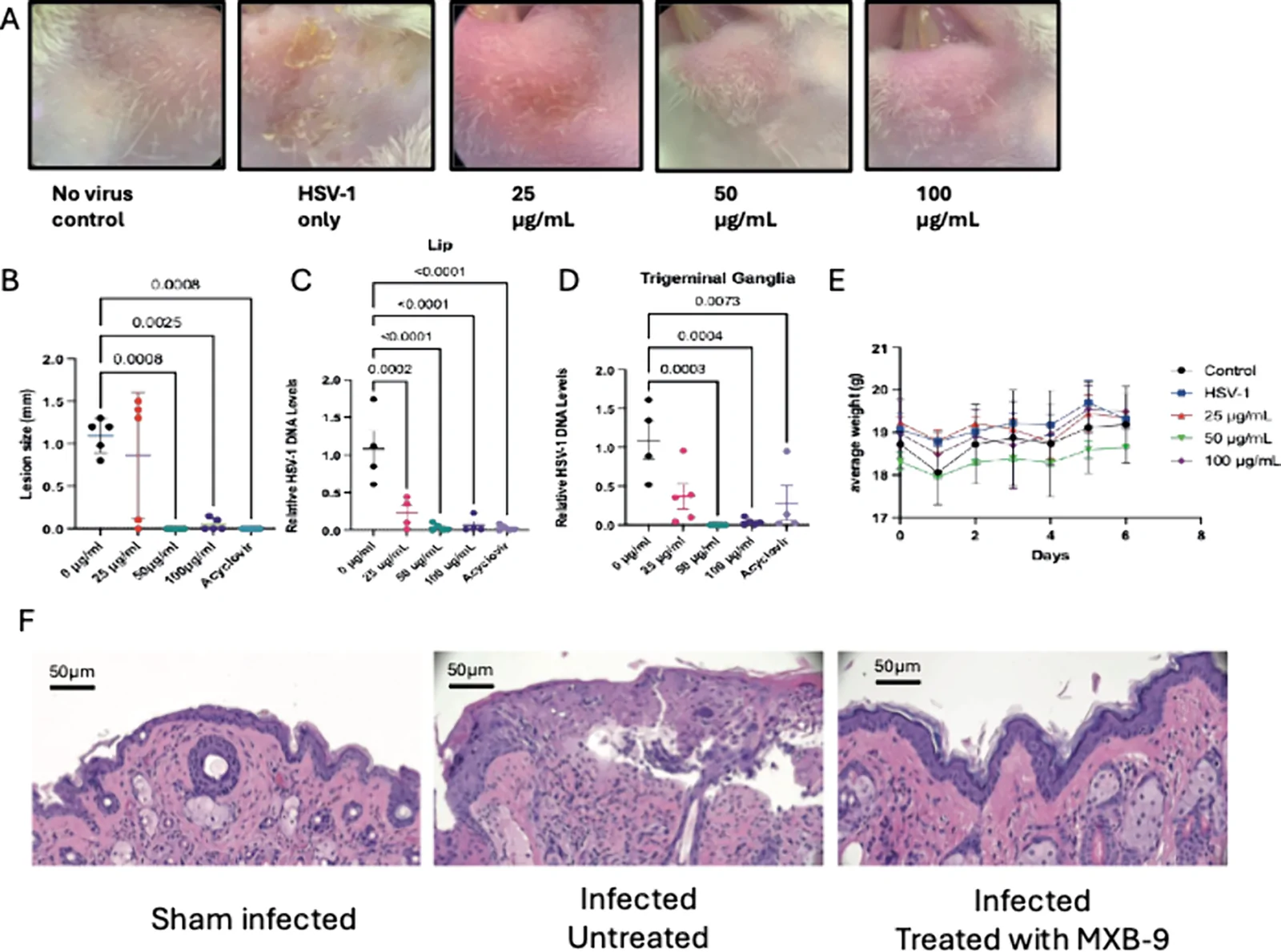
*In vivo* activity of peptoid against HSV-1 in BALB/c mice. BALB/c mice (*n* = 5 per group) were infected with HSV-1 strain 294.1 on day 0 and treated with 20 μL of 0–100 μg/mL of MXB-9 in PBS or 2% acyclovir cream on day 2 postinfection. Lips and TG were harvested on day 5 postinfection for DNA analysis. (A). Photographic images of lesions on day 5. (B) Lesion size on day 5. HSV-1 DNA levels were quantified in excised lips (C) and trigeminal ganglia (D) by qPCR relative to mouse β-actin. Statistical significance was evaluated using One-Way ANOVA and Dunnett’s posthoc test, with error bars representing the standard error of the mean. P-values below 0.05 are provided. Points in B-D represent individual animals. (E) Weight change. Mice were weighed daily. Data show a mean of *n* = 5 ±SD. The experiment was repeated with similar results. (F) Histology. Mice were either sham infected, or infected and untreated or treated with 100 μg/mL MXB-9 as above. At day 5 post infection, lips were excised, fixed and processed for histology. Analysis shows that infected untreated tissue exhibits an ulcerated crust with vesiculation and peripheral epidermal cells showing viral cytopathic effect including ballooning degeneration, chromatin margination, intranuclear inclusions and some multinucleation. Treated tissue shows no viral cytopathic effect or significant inflammatory changes. There are increased mitotic and focal apoptotic figures but no other significant pathologic changes.

Prior to harvest we observed robust lesion clusters in all untreated mice, with lesions spanning the entire lip area (Figure 8A, B). In the 25 μg/mL treated group, lesions were observed in 3 out of 5 mice, while two remained lesion-free. Notably, these lesions exhibited a delayed onset and did not fully develop until day 4 pi. Treatment with 50 μg/mL was highly effective, with no lesion formation observed in any mice and abrasions from the initial infection healing faster without complications overnight. The highest dose also led to healed abrasions, with three mice avoiding lesion development completely, while two mice developed very small lesions between days 4 and 5 (Figure 8B). Treatment with acyclovir led to no visible lesions.

HSV-1 DNA levels in the lip mirrored the lesion images (Figure 8C). A significant decrease in the 25 μg/mL treated group was observed, inhibiting HSV-1 infections by over half compared to untreated (*p* = 0.0368). The 50 μg/mL group exhibited a reduction to nearly undetectable levels, about 90% less than untreated (*p* = 0.0019). The 100 μg/mL group also showed a highly significant (*p* < 0.0001) reduction in viral DNA levels. Viral DNA levels in the trigeminal ganglia were significantly reduced across treatment groups, in a similar dose-dependent manner (Figure 8D). In the 50 μg/mL treated group, the virus was undetectable, showing a 545-fold decrease in viral DNA compared to untreated (*p* = 0.0014). The 100 μg/mL treated group also exhibited a substantial decrease in viral DNA, with levels nearly undetectable (*p* = 0.0017). A similar reduction in viral DNA levels to virtually undetectable was observed with acyclovir treatment. To demonstrate that reduction in viral DNA mirrored reduction in active virus, a parallel sample of mouse lips were homogenized, and viral titer was quantified by TCID_50_ analysis. Untreated lips exhibited a viral titer of 2.15 × 10^4^ TCID_50_/mL, while mice treated with 100 μg/mL there was 1 × 10^1^ TCID_50_/ml, indicating almost complete eradication of infectious virus.

Monitoring of body weight revealed no evidence of weight loss across treatment groups (Figure 8E). Furthermore, no observable signs of localized adverse reactions including redness, peeling skin or swelling were noted at any peptoid concentration or at any point throughout the experimental period. Histological examination of the infected tissue taken at day 5 post infection showed no evidence of viral infection in the lip treated with 100 μg/mL MXB-9 (Figure 8F). Further, there was no significant evidence of pathologic changes due to the peptoid treatment.

## Discussion

Antiviral treatment of oral and labial herpesvirus infections usually involves the use of nucleoside analogues. The reduced efficacy and development of resistance to nucleoside analogues in treating HSV-1 infections demonstrates the need for new antiviral agents that act through different mechanisms. By utilizing a biostable peptidomimetic class of molecules, we have developed novel agents that rapidly inactivate enveloped viruses.^1^ We show here that unlike the acyclovir class of antivirals, as well as the recently described β-carbolines,^20,21^ *in vitro* studies show that antimicrobial peptoids inactivate HSV-1 prior to infection of the target cell. Indeed, when incubated with infected cells, there is little antiviral effect, compared with treatment of uninfected virions. This supports the observations with electron microscopy, that the peptoids disrupt the viral envelope.^1^ Further, using model membrane studies, our prior research demonstrated that this interaction occurs through a mechanism involving association with membrane phosphatidylserine on the outer leaflet.^12^ The results shown here are atypical for standard antiviral agents, such as acyclovir, which primarily interfere with viral replication. Our results indicate that antimicrobial peptoids can directly inactivate extracellular virus prior to infection, thus reducing their infectivity. This activity can further be enhanced with low concentrations of acyclovir. This novel mechanism of action further suggests they may be useful as an adjunct therapy together with antiviral agents that inhibit viral replication.

Numerous mouse models for HSV-1 infection have been developed (reviewed in ref^22^), using a variety of mouse strains as well as viral strains. However, very few are available that specifically recapitulate herpes labialis. To test a new antiviral for efficacy against herpes labialis requires a small animal model that not only provides uniform and reproducible lesions at the site of infection but also recapitulates the translocation to the trigeminal ganglia, without lethal systemic effects. Further, while other models have been shown to provide useful information regarding immune response and other aspects of pathogenesis, none have been optimized specifically for testing topical antiviral drugs on the lips. We examined one model that used a lip scarification technique to compromise the integrity of the epithelium to enhance infectivity into the lip. However, this model used C57Bl/6 mice, which are known to be less susceptible to HSV-1 infection.^18^ Thus, we examined other strains of mice that have been used in other models of HSV-1 infection.^19^ Our results confirmed that C57Bl/6 mice were less susceptible and showed that BALB/c mice were most susceptible to the abrasion model, leading to a highly reproducible lesion with a peak lesion severity at day 5. This allowed us to develop a method to test the efficacy of antiviral peptoids in an early postexposure/early postinfection intervention model. Further, this result provides a useful method to study the pathogenesis of HSV-1 in the development of herpes labialis. It is recognized, however, that this model does not completely mimic the recurrent infection in humans. As discussed below, further development of this model is necessary to provide more robust translational support for research.

Similarly, the strain of virus used to infect the mice is important to obtaining the optimal model. Numerous strains have been used for mouse infections, with varying results.^22^ One consideration was to identify a strain that led to visible lesions but did not lead to lethal infection. We tested several clinical isolates to determine their efficacy in BALB/c mice. Of the strains tested, each possesses unique characteristics relevant to our study. KOS, extensively studied in laboratories, is used in ocular herpes research and is sensitive to acyclovir. HSV-GFP, a recombinant strain of KOS, expresses enhanced green fluorescent protein (EGFP) upon replication, driven by the CMV promoter.^23,24^ This strain could be useful in future real-time *in vivo* imaging studies as our results now demonstrate its pathogenicity. PAAr5, a mutated KOS variant, is resistant to acyclovir.^25^ All of these strains exhibited sensitivity to an antimicrobial peptoid, suggesting that they could all be useful in a test model for drug efficacy. Our results showed that strain 294.1, an acyclovir-sensitive clinical isolate, provided the most optimal lesions and thus was used to test the efficacy of the peptoid *in vivo*. Future studies on the effect of peptoids on reactivation from the TG may examine the possibility of using the McKrae strain, which, while not a clinical isolate, is highly virulent and neuroinvasive and is used in reactivation studies for other sites such as the eyes.

Our *in vitro* results demonstrated an IC_50_ of 8.8 μg/mL for MXB-9, which is close to the IC_50_ of 9.8 μg/mL we observed for LL-37. Thus, we chose this peptoid to test *in vivo* efficacy in this model. Topical administration of MXB-9, when applied daily, beginning on day 2 postinfection demonstrated a dose-dependent inhibition of the development of lesions at the site of infection. Further, there was a similar dose-dependent reduction in the presence of latent virus in the trigeminal ganglia. Treatment with MXB-9 at 50 μg/mL resulted in no lesion formation and a 545-fold reduction in viral load in the trigeminal ganglia compared to the untreated group. Further, the studies showed that the lower concentration of MXB-9 (25 μg/mL) exhibited varying degrees of effectiveness in reducing HSV-1 levels and preventing lesions. This demonstrates that our optimized *in vivo* mouse model represents a robust method for testing topical administration of antivirals for HSV-1 mediated herpes labialis. Further, our results clearly show the potent antiviral activity of peptoids against HSV-1 at this site when delivered 2 days postinfection, comparable to the standard therapy, acyclovir.

However, given that the peptoid was administered topically well after infection, at a time when many cells are already infected, we cannot state unequivocally that the mechanism of action is solely on extracellular virus, as observed in our *in vitro* studies. These could act in concert with direct viral inactivation through membrane disruption to prevent both the development of the lesion and the translocation to the TG.

Significantly, treatment of infected lips with at least 50 μg/mL MXB-9 at day 2 postinfection led to a remarkable reduction in viral DNA in both the lips and trigeminal ganglia over 5 days. Thus, the peptoid may be useful as a prophylactic agent, preventing initial transmission to the lip. The data shown in Figure 2, that the peptoid acts on extracellular virus, suggests that the peptoid is inactivating extracellular virus from the nascent labial infection prior to its entry into adjacent cells. The data from the lip model, however, suggest that it is possible that other mechanisms may also occur when the compound is applied *in vivo*, since the peptoid was applied 2 days postinfection. In addition, since peptoid concentration was not measured in the trigeminal ganglia, further studies could be conducted for antiviral activity on virus that has already translocated to the trigeminal ganglia, where the virus undergoes several rounds of lytic replication.^26^ However, once the virus has achieved latency in the TG, it would be difficult to use the peptoid to prevent reactivation. The development of a reactivation model that leads to recurrent herpes labialis, similar to the fur-plucking model^27^ would be necessary to test the activity of the peptoid against recurrent herpes.

The lack of observable pathology due to the treatment, as well as no effect on weight, suggests that this therapy could be safe for topical use. Given the limited therapeutic options for HSV-1 infections, these findings highlight the potential of peptoids as a robust antiviral treatment, although further testing on the efficacy against already formed lesions and on the rate of lesion resolution needs to be carried out in future studies.

As previously mentioned, compared to peptides, peptoids have the advantage of being resistant to protease degradation^28,29^ with increased stability *in vivo*,^30^ offering an advantage over current antiviral therapies. In general, peptoids are also as easy to scale up and synthesize as peptides.^31^ Additionally, MXB-9 is a short, simple 7-mer peptoid consisting of only four different submonomers, all of which are commercially available. All these factors facilitate scale-up of MXB-9 for future studies.

After testing a variety of common delivery vehicles to eliminate potential inhibitory interactions between the vehicle composition and peptoid activity, we employed PBS without Ca^2+^ and Mg^2+^. However, given the limited ability of PBS to maintain prolonged contact with the tissue, use of a vehicle that allowed increased adherence and longer interaction with the lesion but does not inhibit the activity of the peptoid might permit the use of lower concentrations. While aqueous formulations, such as the PBS utilized in this study, may be adequate, incorporating other widely used excipients like glycerol or propylene glycol could optimize drug delivery to tissues, enhancing the efficacy of the peptoids.

In addition, this study supports the examination of a synergistic approach involving peptoids and existing antiviral agents, such as acyclovir. With further research, this strategic combination could mitigate the risks associated with resistance development while potentially enhancing the treatment efficacy. Furthermore, this approach could alleviate concerns related to the potential toxicity associated with high doses of a single antiviral agent.

## Conclusion

Our results demonstrate that antimicrobial peptoids demonstrate potent *in vivo* activity against HSV-1 in a robust mouse model of herpes labialis when delivered postinfection. This suggests that these compounds are attractive candidates for antiviral agents.

## Methods

### Cell Culture Procedures

Vero E6 cells were obtained from ATCC and were grown and passaged in DMEM/10% FBS at 37 °C, 5% CO_2_. TIGK cells were obtained from Dr. R. Lamont, University of Louisville, and grown and passaged in DermaLife K Keratinocyte Medium (Lifeline Cell Technologies LL-0007).

### Peptoid Synthesis

Starting materials and solvents were purchased from commercial suppliers (Acros Organics, Alfa Aesar, ChemImpex Intl. Inc., CNH Technologies, Merck, OmniSolv, Protein Technologies, Sigma-Aldrich, TCI, and VWR) and used without further purification. Peptoids were synthesized manually using the submonomer method and as previously described.^1,32^ Rink amide MBHA resin 0.64 mmol/g was purchased from Protein Technologies Inc., Tucson, AZ, USA. Substitution with alkylamines was performed in DMF:DCM (1:1 (v/v)) solution overnight. A cocktail of trifluoroacetic acid/triisopropylsilane/water (95:2.5:2.5 (v/v)) was used as cleavage for 30 min. Product purity was determined by means of analytical UPLC/MS using a Water Acquity UPLC system, equipped with an Acquity Diode Array UV detector and a Waters SQD2 mass spectrometer. A Waters Acquity UPLC Peptide BEH C18 Column (300 Å pore size, 1.7 μM particle size, 2.1 mm × 100 mm) with an Acquity UPLC BEH C18 VanGuard precolumn (1.7 μM, 2.1 mm × 5 mm) was employed as stationary phase. Purification, by means of preparative HPLC, was carried out using a Waters Prep150LC system, equipped with a Waters 2489 UV/Visible detector and a Waters Fraction Collector III collector, and a Waters XBridge BEH300 Prep C18 column (5 μM particle size, 19 mm × 100 mm) with a Waters XBridge Peptide BEH300 C18 guard column (5 μM particle size, 19 mm × 10 mm). After purification, exchange of the counterion was carried out using a 10 mM solution of aqueous HCl. Lyophilization yielded the desired compound with the purity higher than 95%.

### Viral Strains

HSV-1 strains PAAr5, TKR, KOS, 615.8D, 615.9, and 294.1 were obtained from D. Don Coen, Harvard University. HSV-1 GFP was obtained from Dr. K. Gus Kousoulas (Louisiana State University).^23^ All strains were propagated, and the titer was quantified as described in ref.^24^

### Susceptibility of Viral Strains to Peptoids In Vitro

Viral strains (10^5^ pfu) were treated with peptoid at 20 μg/mL for 2 h in a volume of 100 μL in PBS. One microliter of this was used to infect a well of TIGK cells (2 × 10^5^ cells). After a 24 h incubation, total DNA was isolated, and viral DNA was quantified by QPCR as described.^1^ Specifically, DNA extraction was performed on each well using the Macherey-Nagel (Allentown, PA, USA) NucleoSpin and DNA RapidLyse Kit, per the manufacturer’s stipulated guidelines. Quantification of HSV-1 DNA levels was carried out by quantitative PCR (qPCR) relative to β-actin as described^1^ on a BIO RAD myCycler with SsoAdvanced Universal SYBR green supermix. Primers for HSV-1 UL30 were F: AGAGG­GACAT­CCAGG­ACTT­TGT; R: CAGGC­GCTTG­TTGG­TGTAC; β-Actin primers were F: GGATC­AGCAA­GCAGG­AGTATG; R: AGAAA­GGGTG­TAACG­CCAC­TAA. Relative HSV-1 DNA levels were determined using the 2^–ΔΔCt^ method compared to untreated samples. To determine viral titers, TCID_50_ was performed in VERO E6 cells seeded in 96-well plates. HSV-1 was serially diluted 1:10 in media to create 8 dilutions. 100 μL of diluted viruses was added to cells (in quadruplicate) and incubated for 72 h. CPE formation in wells was recorded and used to determine the TCID_50_ as described.^33^ All experiments were carried out in triplicate and repeated at least once.

### Identification of Peptoid Activity in the HSV-1 Life Cycle

TIGK cells were seeded in a 24-well plate and grown to 70–80% confluence (2 × 10^5^ cells). Cultures were subjected to infection with HSV-1 GFP at a multiplicity of infection (MOI) of 0.1:1 at 4 °C. Following the infection stage, wells underwent two washes using fresh medium, after which fresh medium was introduced at 37 °C, followed by an incubation lasting 30 min. At different points in the infection, triplicate cultures were treated with MXB-9 at 20 μg/mL.

The treatments were as follows: (1) the introduction of virus without peptoid treatment (“virus only”), (2) application of peptoid to virus prior to infection (“pre-attachment”), (3) peptoid introduction during the 4 °C incubation phase (“attachment”), (4) peptoid inclusion during the 37 °C 30 min incubation phase (“penetration”), and (5) peptoid addition post citrate buffer wash (“post entry”). Following the buffer wash, wells were subjected to two rinses with fresh medium.

### In Vivo Mouse Model of Infection and Peptoid Treatment

Female mice (BALB/C, C57Bl/6, AJ, and SJL/J), aged 6–8 weeks, were procured from Jackson Laboratories (Bar Harbor, ME). The study adhered to protocols approved by the Institutional Animal Care and Use Committee (IACUC) at the University of Louisville (protocol number 20808). Power analysis^34^ was carried out based on preliminary experiments, to obtain an optimal group size of *n* = 5.

Anesthesia was administered to the mice using ketamine/xylazine (50 mg/kg ketamine/10 mg/kg xylazine), and a visible abrasion was created on the lip through gentle scraping with a scalpel blade. Subsequently, virus (5 × 10^7^ pfu/mL or Vero cell lysate) was topically applied in 10 μL, and mice were allowed to remain sedated for a further 30 min. On day 2 post infection, mice were treated with either LL-37 or MXB-9 diluted in PBS, topically administered to the surface of lip in a 20 μL volume. As a positive control, acyclovir (Zovirax, 5%, IsraelPharm) was applied to the lips in parallel. The mice were kept under isoflurane anesthesia for 5 min to facilitate drug penetration and to prevent removal of the vehicle. Weights were determined daily, and lesion size measurements through photomicrography were conducted daily. Furthermore, overall adverse effects of both viral infection and treatments were conducted by observing general animal health and behavior by two separate investigators.

To quantify infection, the mice were euthanized by CO_2_, and the lip lesions and trigeminal ganglia were excised. Tissue homogenization was carried out using Macherey-Nagel MN Bead Tubes Type D for DNA isolation Macherey-Nagel NucleoSpin, Mini kit. HSV-1 DNA was quantified by qPCR relative to murine β-actin as described above, and plaque assays were also carried out.^24^ Statistical significance was evaluated via ANOVA with Dunnett’s multiple comparison posthoc testing (*p* < 0.05). Experiments were repeated with similar results.

To assess the effects on the tissue, lips were excised on day 5 postinfection, fixed in formalin, and processed for histology. H&E-stained slides were examined by an oral pathologist, blinded to the experimental conditions.

### Statistical Methods

Experiments were analyzed using GraphPad Prism 10 with unpaired *t* tests, one and two-way analysis of variance (ANOVA) with Dunnet’s posthoc test for multiple comparisons. Normality was determined by Kolmogorov–Smirnov test.

## ¶

Chief Scientific Officer, Curio Wellness, Towson, MD 21285, United States
